# Metagenomic Analyses of Gut Bacteria of Two Sandfly Species from Western Ghats, India, Differing in Their Vector Competence for Leishmaniasis

**DOI:** 10.3390/microorganisms13071615

**Published:** 2025-07-09

**Authors:** Anns Tom, Nanda Kumar Yellapu, Manju Rahi, Prasanta Saini

**Affiliations:** 1 ICMR-Vector Control Research Centre, Puducherry 605006, India; annsmary25@gmail.com (A.T.); nandakumaryellapu@gmail.com (N.K.Y.); drmanjurahi@gmail.com (M.R.); 2 School of Life Sciences, Pondicherry University, Kalapet, Puducherry 605014, India

**Keywords:** leishmaniasis, *Phlebotomus argentipes*, *Sergentomyia babu*, gut microbiota, metagenomics

## Abstract

Phlebotomine sandflies are the primary vectors of *Leishmania* parasites, the causative agents of leishmaniasis. In India, *Phlebotomus argentipes* is the confirmed vector of *Leishmania donovani*. The sandfly gut microbiota plays a crucial role in *Leishmania* development and transmission, yet it remains largely understudied. This study used a metagenomic approach targeting the V3–V4 region of the 16S rRNA gene to compare the gut bacterial communities of *P. argentipes* and *Sergentomyia babu* prevalent in Kerala. A total of 18 distinct bacterial phyla were identified in *P. argentipes*, and 14 in *S. babu*, both dominated by *Proteobacteria*, *Actinobacteria*, and *Firmicutes*. A total of 315 genera were identified in *P. argentipes*, with a high relative abundance of *Pseudomonas* (6.3%), whereas *S. babu* harbored 327 genera, with *Pseudomonas* showing a higher relative abundance of 11%. Unique to *P. argentipes*, bacterial phyla such as Fusobacteria, Armatimonadetes, Elusimicrobia, Chlamydiae, and Crenarchaeota were identified, whereas Chlorobi was specific to *S. babu.* Additionally, 145 species were identified in *P. argentipes*, compared to 164 species in *S. babu*. These findings provide a comparative baseline of gut microbial diversity between vector and non-vector sandfly species, offering a foundation for future functional investigations into vector competence.

## 1. Introduction

Leishmaniasis is a complex, multifaceted tropical and subtropical disease caused by *Leishmania*, an obligate digenetic protozoan parasite. It affects millions of people worldwide [1]. Phlebotomine sandflies have been recognized as the prominent insect vectors of leishmaniasis, owing to their ability to carry and transfer *Leishmania.* Numerous incidences of both cutaneous leishmaniasis (CL) and visceral leishmaniasis (VL) have been reported over the past two decades in Kerala, one of the southern states of India [2,3]. According to epidemiological records, leishmaniasis is prevalent among tribal settlements and villages near the Western Ghats in Kerala, where the tropical climate, preserved forest environment, limited human presence, and humid, shady microenvironments facilitate the breeding and spread of the disease [3].

Among phlebotomine sandflies, *Phlebotomus argentipes* is considered as the principal vector for leishmaniasis in the Indian subcontinent, being accountable for the majority of fatal visceral leishmaniasis and cutaneous leishmaniasis instances in the region caused by *L. donovani* [2,4], while the role of *S. babu* in *Leishmania* transmission in humans is unproven. Members of the genus *Sergentomyia* have widespread distribution across the Old World, especially in the Indian subregion, and some are suspected vectors of *Leishmania*, owing to the presence of *Leishmania* DNA [5]. *Sergentomyia* can only be confirmed as a vector for *Leishmania* transmission if certain criteria are met. These include repeated natural infection with the same *Leishmania* species in humans and reservoir hosts, exhibit preference for feeding on humans, and in cases of zoonotic transmission, must also feed on reservoir hosts, establishing strong ecological associations between the vector, humans, and reservoir hosts, and possess the ability to support the parasite’s complete development [6,7].

The sandflies’ gut microbiome plays a crucial role in parasite’s survival, proliferation, and transmission, while the gut microbiota is shaped by factors such as host species, geographic location, and environmental conditions [8,9]. A notable correlation exists between the unique composition of microbial gut flora and the geographical area inhabited by the sandfly, highlighting the ecological conditions pertinent to their habitat [10]. Recent studies demonstrate the complex relationship between nutrition, sandfly development, and microbiome composition [11]. Understanding the difference in gut bacterial communities between *P. argentipes* and *S. babu*, can provide significant insights into the microbiome-mediated mechanisms of vector competence, as gut microbiota are increasingly recognized for their role in modulating pathogen transmission dynamics [10]. These microbial communities are influenced by developmental stages, host–habitat interaction, and food sources acquired from the surrounding environment, including plants and animal hosts [11,12]. Certain gut bacteria can act as natural barriers to *Leishmania* by producing digestive enzymes that kill the parasites [13,14,15], while others may create a more permissive environment for the parasite’s survival and proliferation [16]. Many of the earlier studies on sandfly gut microbiota studies are culture-based [17,18,19], having identified the prevalence of members of the phylum *Proteobacteria* and *Firmicutes* in *P. argentipes* gut [19]. However, these approaches are limited to bacteria that can grow under laboratory conditions and potentially losing a significant portion of the microbiome [20]. On the contrary, metagenomics provides a more comprehensive, culture independent analysis revealing a broader microbial composition without prior assumptions of bacterial community makeup [21,22].

In this study, we utilized a metagenomic approach to compare the gut bacterial diversity in female gravid *P. argentipes*, a primary *Leishmania* vector, and *S. babu*, a non-vector species. Female sandflies play a pivotal role in disease transmission, particularly due to their hematophagous behavior exposing them to various pathogens, including bacteria. The diverse diet of female sandflies, including blood and other substances, significantly influences their gut microbiota, offering insights into the complex interactions between microbiota and parasite transmission [8,9]. The sandfly gut microbiome can significantly impact vector competence for *Leishmania* transmission [10,12]. Metagenomic studies also facilitate insights into the interactions of *Leishmania* parasites and the microbiota of sandflies and are vital for developing para-transgenic strategies to control disease transmission [23].

## 2. Materials and Methods

### 2.1. Sample Collection

In this study, sandflies were collected from Kottayam district (9°32′24.2″ N 76°37′46.1″ E) in Kerala. Sandfly specimens were collected using a combination of mouth aspirators, mechanical aspirators, and CDC-modified light traps. For optimal sample collection, 4–5 sites were selected, considering favorable environmental conditions. Light traps were strategically positioned 1–1.5 m above the ground to capture sandflies between 6:00 p.m. and 6:00 a.m. Daytime collection, from 8:00 a.m. to 6:00 p.m., involved the use of mechanical aspirators and mouth aspirators. Sandflies were mainly collected from cattle sheds and houses indoors.

### 2.2. Processing and Identification of Field-Collected Sandflies

The field-collected living sandflies were transported to the ICMR-VCRC field station in Kottayam and carefully maintained in sandfly cages provided with 30% sucrose solution. Living sandflies were first immobilized on ice, and only gravid females were chosen and surface sterilized by washing with 70% ethyl alcohol, followed by thorough washing in PBS (1X) three times to eliminate residual traces of ethyl alcohol externally. Then, mouth parts, wings, legs, and the final three abdominal segments were dissected using sterile microneedles and permanently mounted on microscope slides in Hoyer’s media for future reference. Specimens were morphologically identified under a compound microscope (Olympus CX41) based on standard keys by [24,25,26]. The aseptically dissected gut of each specimen was incubated in individual tubes in 10 µL of PBS (1X) and stored at −80 °C [19]. A total of 70 guts of each gravid females of *P. argentipes* and of *S. babu* were pooled separately and homogenized.

### 2.3. DNA Extraction, PCR Amplification, and 16S rRNA Sequencing

Genomic DNA was extracted using a conventional Phenol Chloroform method [27,28] and used as a template for PCR amplification of the V3–V4 region of the 16S rRNA gene using gene-specific primers 341F (CCTAYGGGRBGCASCAG) and 806R (GGACTACNNGGGTATCTAAT). PCR amplification was performed with incubation at 98 °C for 1 min, followed by 30 cycles of incubation at 98 °C (10 s), 57 °C (30 s), and 72 °C (1 min), with a final extension cycle of 5 min at 72 °C [29]. The amplicons obtained from the PCR reaction were purified and checked with the Bioanalyzer 2100 (Agilent Technologies, Santa Clara, CA, USA). Libraries were prepared using the TruSeq DNA PCR-Free Library Preparation Kit (Illumina, San Diego, CA, USA). The prepared libraries were quantified using a Qubit 4 fluorometer (Thermo Fisher Scientific, Waltham, MA, USA) and Quant Studio 5 real-time PCR (Applied Biosystems, Waltham, MA, USA). The qualified libraries were sequenced using paired-end chemistry on the NovaSeq 6000 platform (Illumina, USA) with a read length of 250 bp.

### 2.4. Bioinformatic Analysis of Amplicon Sequencing Data

The raw sequence data were subjected to multiple levels of quality filtering. Initially, FastQC was used for quality control, followed by Trim Galore for 3’-end trimming and adapter removal. The trimmed sequences were then processed for taxonomic classification. Unlike traditional Operational Taxonomic Unit (OTU)-based approaches, we employed Kraken2 (v2.1.2) as the primary taxonomic classification tool with a combined Silva (v138.1)- and Greengenes (v2022)-customized database. This approach directly generates taxonomic assignments (TAXA) rather than clustering sequences into OTUs, providing a more accurate representation of the microbial community structure [30,31,32]. The taxonomic data and abundance tables were exported for downstream analyses in R (v4.2.2). The Phyloseq package (v1.42.0) was used to generate a Phyloseq object from the Kraken2 taxonomic classification outputs. For visualization of the microbiome composition, Krona Tools (v2.8.0) were used to generate interactive Krona charts displaying the hierarchical taxonomic structure of the bacterial communities.

Alpha diversity analysis was applied to evaluate the complexity of species diversity within each sample through indices such as observed taxa, Chao1, ACE (Abundance-based Coverage Estimator), Shannon, Simpson, Inverse Simpson, and Fisher’s alpha using the Vegan R package (v2.6-4). Rarefaction curves were also generated to assess sampling depth sufficiency. Beta diversity was analyzed using Bray–Curtis dissimilarity to evaluate the differences in species complexity between samples. The ggplot2 R package (v3.4.1) was utilized for abundance plotting and visualization of diversity metrics. For data presentation and visualization, the Tidyverse (v2.0.0) and ggplot2 packages were employed to ensure clean and organized display of results.

## 3. Results

### 3.1. Next-Generation Sequencing (NGS) Data of Gut Bacteria of P. argentipes (PAG-1) and S. babu (SBG-2)

The Next-Generation Sequencing approach was employed to effectively characterize the bacterial microbiome of gravid female sandflies of *P. argentipes* and *S. babu* collected from the study areas. In total, 700,002 quality-filtered reads were obtained from the V3–V4 region for both *P. argentipes* and *S. babu.* Following taxonomic classification with Kraken2 using the combined Silva and Greengenes reference database, these reads were assigned to 1270 distinct taxa. The total of 378,038 raw reads detected in *P. argentipes* yielded 629 distinct taxonomic assignments, while *S. babu* yielded 321,964 reads that generated 641 taxonomic assignments. The total number of bases obtained for *P. argentipes* was 113,789,438 and 96,911,164 for *S. babu*. The average read length for both samples was 301 bp. The GC content of both samples was found to be 55%. The read quality score observed for *P. argentipes* was 32.75 and 32.81 for *S. babu* (Table 1). The sequencing data have been submitted to the NCBI Sequence Read Archive (SRA) under the BioProject accession number PRJNA1266154, with an individual accession number SRR33657226 for *P. argentipes* and SRR3365225 for *S. babu*.

### 3.2. Taxonomic Composition of Gut Bacteria

**Figure 1:**
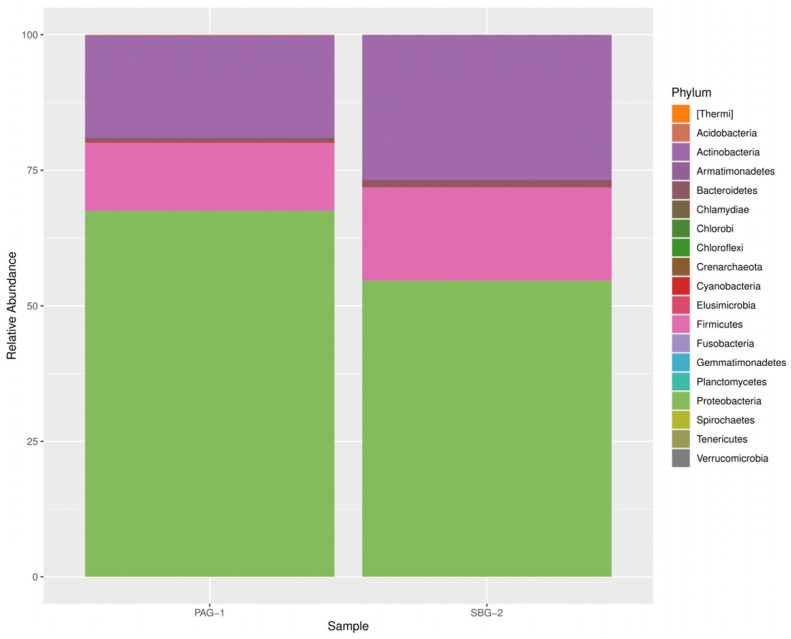
Relative abundance of bacterial phyla in *P. argentipes* and *S. babu*. The taxa are indicated by their respective color. The bar plot displays only the most abundant bacterial phyla, while less abundant phyla are grouped or omitted for clarity.

A total of 18 distinct bacterial phyla were identified in *P. argentipes* with *Proteobacteria*, *Actinobacteria*, and *Firmicutes* being the most dominant. *Proteobacteria* represented the most abundant phylum within the gut microbiota of gravid female sandflies. Similarly, *S. babu* exhibited 14 bacterial phyla, demonstrating a comparable dominance pattern (Figure 1).

Among the gut bacteria in *P. argentipes*, a total of 41 classes, 100 orders, 181 families, 315 genera, and 145 species were identified. In contrast, *S. babu* revealed 40 classes, 96 orders, 108 families, 327 genera, and 164 species (Table 2).

**Figure 2:**
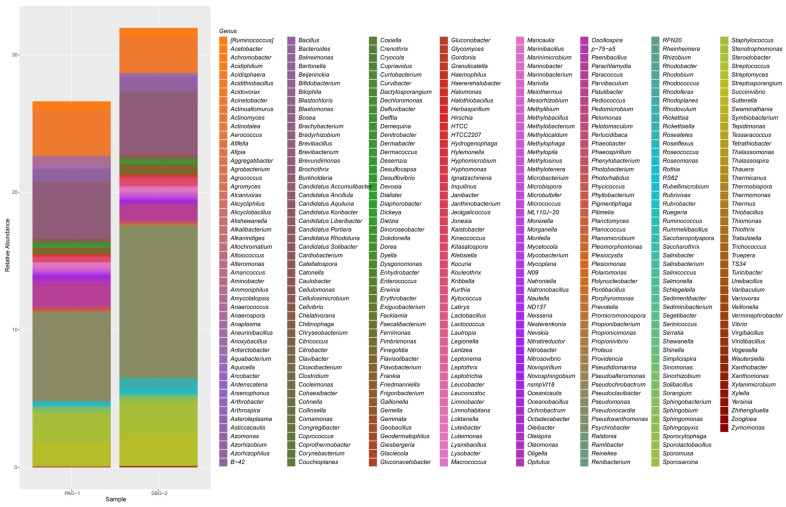
Stacked bar plot showing the relative abundance of bacterial genera in *P. argentipes* (PAG-1) and *S. babu* (SBG-2) gut microbiota. The height of each colored segment represents the proportional abundance of individual bacterial genera, allowing for direct comparison between the two sandfly species.

Both *P. argentipes* and *S. babu* exhibited a dominance over a few bacterial classes such as Alphaproteobacteria (41.5% in *P. argentipes*; 25% *S. babu*), Gammaproteobacteria (24% in *P. argentipes*; 27.4% in SBG-2), Actinobacteria (19% in *P. argentipes*; 27% in *S. babu*), and Bacilli (12.4% in *P. argentipes*; 17% in *S. babu*). These classes collectively accounted for approximately 80% of the bacterial communities in both species. Minor classes, such as Betaproteobacteria and Erysipelotrichia, were present at low levels. Among the dominant bacterial orders, Caulobacterales (27% in *P. argentipes*; 15% in *S. babu*), Actinomycetales (19% in *P. argentipes*; 26% in *S. babu*), Bacillales (12.2% in PAG-1; 17% in *S. babu*), and Pseudomonadales (15% in both *P. argentipes* and SBG-2) were prevalent. At the bacterial family level, *P. argentipes* exhibited a dominance of Enterobacteriaceae (18%), Bacillaceae (12%), and Pseudomonadaceae (10%). But *S. babu* was dominated by Lactobacillaceae (20%), Micrococcaceae (15%), and Spiroplasmataceae (10%). At the genus level, in *P. argentipes*, *Streptococcus* showed the highest relative abundance at 16%, followed by *Lysinibacillus* (13%), *Brevibacterium* (9.4%), and *Pseudomonas* (7%). In contrast, in *S. babu*, *Actinobacteria* showed the highest relative abundance at 25.3%, followed by *Streptococcus* (22.18%), *Pseudomonas* (19%), *Brevundimonas* (8.3%), and *Lactobacillus* (2.23%) (Figure 2). At the species level, in *P. argentipes*, bacterial species such as *Brevundimonas diminuta*, *Lysinibacillus boronitolerans*, *Bacillus flexus*, *Stenotrophomonas geniculata*, *Phaeobacter gallaeciensis*, *Acinetobacter johnsonii*, *Staphylococcus aureus*, *Paenibacillus stellifer*, and *Sphingobacterium multivorum* were dominantly present. In *S. babu*, species such as *Paracoccus aminovorans*, *Brevundimonas diminuta*, *Lysinibacillus boronitolerans*, *Roseomonas mucosa*, *Staphylococcus aureus*, *Bacillus flexus*, *Paenibacillus stellifer*, *Anoxybacillus kestanbolensis*, and *Sphingobacterium multivorum* were dominant (see Supplementary Figure S1). Taxonomic profiles were visualized using Krona plots (see Supplementary Files S1 and S2 for *P. argentipes* and *S. babu*, respectively).

**Figure 3:**
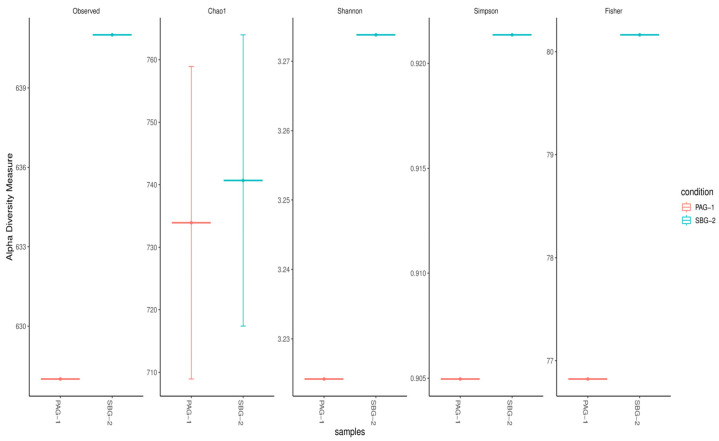
Alpha diversity indices comparing bacterial gut microbiota of *P. argentipes* (PAG-1) and *S. babu* (SBG-2). Diversity was assessed using five metrics: Observed, Chao1, Shannon, Simpson, and Fisher. *S. babu* exhibited higher species richness across all indices, indicating a more diverse microbial community than *P. argentipes*.

Diversity indices, including alpha and beta diversity, were used to compare the bacterial gut microbiota of *P. argentipes* and *S. babu*. Alpha diversity was calculated based on five metrics: Observed, Chao1, Shannon, Simpson, and Fisher indices (Figure 3). The Observed taxa and Chao1 indices indicated that approximately 633 species were found in *P. argentipes*, while 760 species were observed in *S. babu*. The Shannon index showed higher evenness in *S. babu*, representing a more balanced bacterial community. Therefore, *S. babu* exhibited greater species richness compared to *P. argentipes*, indicating a more diverse microbial community in terms of the number of species present.

**Figure 4:**
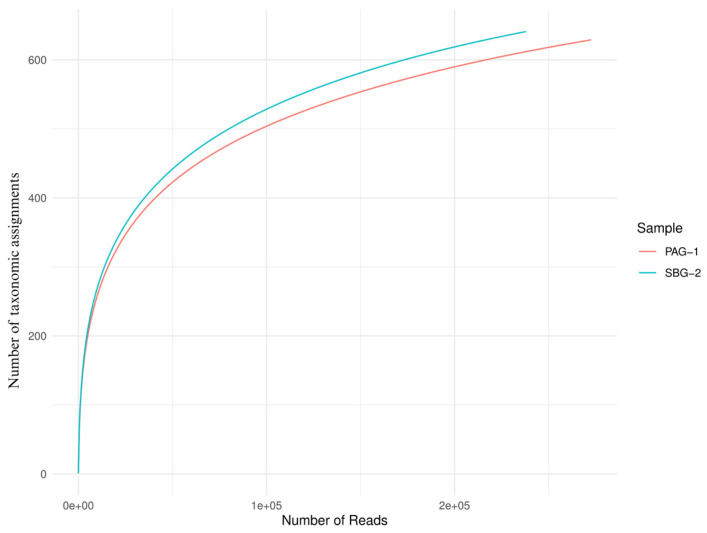
Rarefaction curves of *P. argentipes* (PAG-1) and *S. babu* (SBG-2) gut microbiota. The x-axis represents the number of raw reads sampled, while the y-axis shows the number of unique taxonomic assignments observed. The blue curve (SBG-2) reaches a higher plateau than the red curve (PAG-1), demonstrating that SBG-2 harbors greater microbial diversity at equivalent sampling depths.

**Figure 5:**
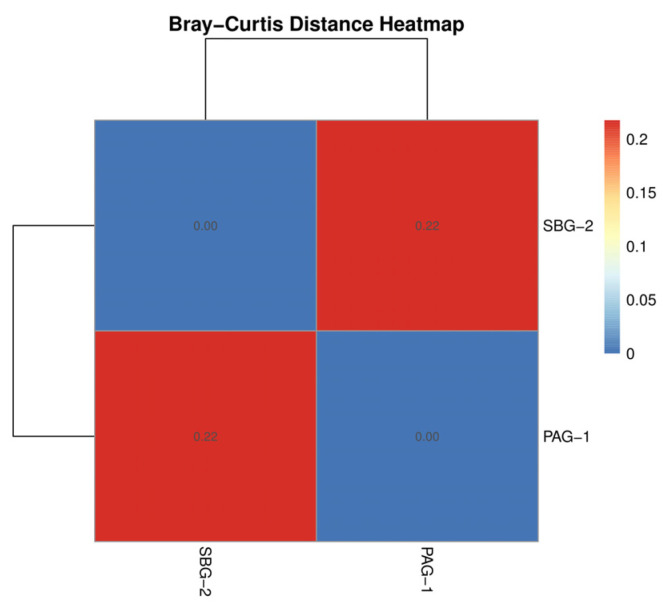
Bray–Curtis similarity heatmap comparing gut microbiota compositions of *P. argentipes* (PAG-1) and *S. babu* (SBG-2). The color gradient ranges from red (lower similarity, higher dissimilarity) to blue (higher similarity, lower dissimilarity), as indicated by the color scale bar.

Alpha diversity metrics (Observed, Chao 1, Shannon, Simpson, Fisher; Figure 3) and rarefaction curves (Figure 4) both indicate that *S. babu* (SBG-2) harbors greater species richness and evenness than *P. argentipes* (PAG-1), while Bray–Curtis beta diversity analysis (Figure 5) reveals lower community similarity in SBG-2, supporting its overall higher microbiome diversity.

## 4. Discussion

In recent years, there has been increased effort to study gut microbiota of insect vectors, particularly those with medical significance such as mosquitoes and sandflies [33]. The composition of bacterial communities in these vectors plays a crucial role in their competence to transmit pathogens. For example, the presence of *Wolbachia* in mosquitoes has been shown to reduce the ability to transmit viruses such as Dengue and Zika by inducing cytoplasmic incompatibility and activating host immune responses [34]. Similarly, *Chromobacterium* spp. have been implicated in suppressing the transmission of malaria and dengue viruses by mosquitoes [35]. Symbiotic bacteria such as *Sodalis glossinidius*, *Spiroplasma* sp. and *Wolbachia* have also been shown to hinder *Trypanosoma grayi* coexistence in wild population of tsetse flies [36]. In sandflies, the gut microbiome is intricately linked to host physiology and vectorial capacity. The pathogenicity of the *Leishmania* parasite depends on successful progression through specific developmental stages within the sandfly midguts [15]. In several studies, the microbiota residing in the gut of insect vectors has been shown to constitute a specialized niche that facilitates accelerated microevolutionary processes [8,9,14]. However, majority of studies conducted to date have relied on culture-based methods, which inherently limit the identification of non-culturable bacterial taxa.

This study represents the first metagenomic approach used to investigate the gut microbiota of *P. argentipes* and *S. babu*. The observed interspecies differences in microbial composition underscore the importance of considering both vector and non-vector species in microbiome research to discern their potential roles in modulating vector competence and disease transmission. Although previous studies have explored the gut microbiota of sandflies across geographic regions [37], most have focused exclusively on environmental factors without comparing vector and non-vector species [17,37]. In sandflies, prior evidence suggests that midgut bacteria can influence *Leishmania* metabolism and virulence [38,39]. To address this knowledge gap and establish comparative baseline data, we conducted a metagenomic analysis targeting the V3–V4 hypervariable regions of the 16S rRNA gene in the midgut microbiota of both *P. argentipes* (vector) and *S. babu* (non-vector) species.

**Figure 6:**
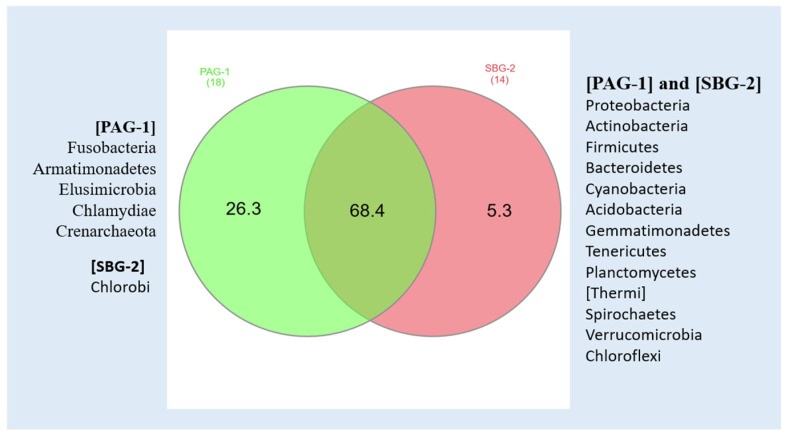
Venn diagram showing bacterial phyla in *P. argentipes* (PAG-1) and *S. babu* (SBG-2), highlighting taxa exclusive to each species and those shared between them.

Our results identified 629 taxonomic assignments in *P. argentipes* and 641 in *S. babu*. This approach achieved complete phylum level identification in both species. Approximately 26% of bacterial sequences in *P. argentipes* and 32% in *S. babu* were classified at the genus level. Notably, *P. argentipes* exhibited a higher phylum diversity (18 phyla) compared to *S. babu* (14 phyla). Phyla such as Fusobacteria, Armatimonadetes, Elusimicrobia, Chlamydiae, and Crenarchaeota were unique to *P. argentipes* (26.3%), whereas phylum Chlorobi (5.3%) was exclusive to *S. Babu.* A total of 13 phyla were shared between both species including Proteobacteria, Actinobacteria, Firmicutes, Bacteroidetes, Cyanobacteria, Acidobacteria, Gemmatimonadetes, Tenericutes, Planctomycetes, Thermi, Spirochaetes, Verrucomicrobia, and Chloroflexi. This overlap (68.4%) likely reflects their shared environment and similar ecological niche, as both species were collected from the same geographical location (Figure 6).

In the present study, Proteobacteria constituted the dominant bacterial phylum in both species, comprising approximately 65% of the total relative abundance. This finding corroborates a previous study by Gunathilaka et al. [19], who documented a high prevalence of Proteobacteria in *P. argentipes* using culture-dependent methodologies. Proteobacteria in the sandfly gut microbiome are significant, as members of this phylum can contribute to the nutritional and metabolic requirements of their insect hosts [40], thereby enhancing their survival through nitrogen fixation and other metabolic functions. Actinobacteria ranked as the second most prevalent phylum in both sandfly species (approximately 22%), a finding that contrasts with previous investigations in which Firmicutes were reported as the second most prevalent phylum [18]. The third most abundant phylum was Firmicutes (approximately 9%), followed by Bacteroidetes and Tenericutes. These findings indicate potential geographical and species-specific differences in gut microbiota composition.

We hypothesized that microbial exclusivity might influence vectorial capacity in *P. argentipes*. Notably, Fusobacteria, Armatimonadetes, Elusimicrobia, Chlamydiae, and Crenarchaeota were specific to *P. argentipes*. Fusobacteria, a phylum of Gram-negative bacteria, is generally associated with pathogenicity in mammals and could contribute to host–parasite interactions [41]. Members of the Armatimonadetes are typically associated with soil, water, and gut environments of animals and insects usually involved in nutrient cycling and degradation of organic matter [42]. Elusimicrobia is a predominantly anaerobic group often associated with the fermentation of complex carbohydrates. Members of the Chlamydiae are known for their pathogenic potential in humans and animals. These bacteria can produce antimicrobial compounds such as bacteriocins and antimicrobial peptides (AMPs), potentially inhibiting the growth of competing microorganisms and create a more favorable environment for *Leishmania* survival. In contrast, *S. babu* uniquely harbored Phylum Chlorobi. These anoxygenic green sulfur bacteria provide nutritional support to their insect hosts through their metabolic byproducts.

Genus-level analysis identified 315 genera in *P. argentipes* and 327 in *S. babu*. Despite hosting a smaller number of phyla compared to *P. argentipes*, *S. babu* exhibited higher genus richness. A total of 17.4% of genera were unique to *P. argentipes* and 20.5% of genera were unique to *S. babu*, while 61% were commonly shared. Genera such as *Pseudomonas* (6.3%), *Acinetobacter* (3.2%), *Brevibacterium* (3%), *Streptomyces* (2%), *Stenotrophomonas* (2%), *Paracoccus* (2%), and *Bacillus* (1%) showed high relative abundance in *P. argentipes*. In contrast, *S. babu* exhibited higher relative abundance for genera such as *Pseudomonas* (11%), *Brevibacterium* (4%), *Acinetobacter* (3%), *Streptomyces* (2%), *Stenotrophomonas* (1%), *Bacillus* (1%), *Staphylococcus* (1%), and *Rickettsia* (1%). In *P. argentipes*, 69 unique bacterial genera were identified. Among them, *Candidatus* showed maximum relative abundance (0.085%), followed by *Saccharopolyspora* (0.05%), *Sporomusa* (0.04%), etc. Similarly in *S. babu*, 81 unique genera were identified. Among them, *Serinicoccus* (0.02%), *Kytococcus* (0.01%), and *Meiothermus* (0.01%) showed the highest relative abundance, though their values were very low.

Similar analysis of the gut microbiota of *S. babu* also seems to be important to understand the microbial factors contributing to its non-vector status. The detection of *Rickettsia* in the midgut of sandflies raises intriguing questions about the potential interactions with other microbial inhabitants and their cumulative effect on the sandfly and *Leishmania* parasites. In *S. babu*, *Rickettsia* was detected with a total of 1668 classified reads, compared to only 4 in *P. argentipes*. The presence of *Rickettsia*, an endosymbiont or facultative symbiont of insects, can influence vector physiology, immune responses, and overall insect health, potentially affecting its competency as a vector for *Leishmania* transmission by modifying the microbial environment and immune mechanisms [43]. Therefore, it can be inferred that the elevated immune response and improved health of the sandfly may create an unsuitable environment for the survival of *Leishmania* in the gut, contributing to the incompetence of *S. babu.* Interestingly, common insect-specific endosymbionts such as *Wolbachia*—whose presence was already detected in sandfly gut—*Spiroplasma*, and *Sodalis* were not detected in both *P. argentipes* and *S. babu*. Previous studies have documented *Rickettsia* in various insect species, including *P. chinensis* and several mosquitoes (*Anopheles*, *Culex*, and *Aedes*) across regions such as China [44], Africa [45], and the USA [46], indicating a broader ecological interaction that warrants further investigations. While this descriptive study establishes important differences in bacterial communities between vector and non-vector species, experimental functional studies will be necessary to determine the exact role of these bacteria in modulating the transmission and survival of *Leishmania* in the sandfly gut. Moreover, *Sergentomyia* species primarily feed on cold-blooded vertebrates, specifically reptiles [47]. Thus, their feeding preference for reptilian hosts rather than mammals limits their potency to transmit human leishmaniasis.

Apart from this, many of the identified bacteria are pathogenic to humans and animals and are commonly present in both sandfly species. Some genera include *Acinetobacter*, *Bacillus*, *Clostridium*, *Haemophilus*, *Klebsiella*, *Mycobacterium*, *Neisseria*, *Proteus*, *Pseudomonas*, *Rickettsia*, *Salmonella*, *Serratia*, *Staphylococcus*, *Vibrio*, and *Yersinia*. In the case of *S. babu*, approximately eight genera including *Rickettsia*, *Serratia*, *Pseudomonas*, *Acinetobacter*, *Staphylococcus*, *Bacillus*, *Clostridium*, and *Proteus* were identified as pathogenic.

In alpha and beta diversity analysis, *S. babu* possesses higher microbial diversity, richness, and evenness compared to *P. argentipes*. This higher diversity may reflect ecological and physiological differences between the two sandfly species. Similarly, the distinct clustering observed in the beta diversity analysis highlights the influence of host-specific factors on shaping gut microbiota. Metagenomic studies particularly utilizing the V3–V4 region of the 16S rRNA gene provide superior insights compared to culture-based studies. The impact and interactions of these significantly large, unidentified bacteria remain unknown. Additionally, this study focused solely on bacterial components, neglecting the potential influence of viruses, fungi, and protozoa on *Leishmania* survival and transmission.

## 5. Conclusions

The composition of an insect gut microbiome significantly influences its capacity to transmit diseases. This study presents the first comprehensive metagenomic comparative analysis of the gut microbiota in the *P. argentipes*, a confirmed vector of *Leishmania donovani*, and *S. babu*, a non-vector species. We identified noble differences in bacterial diversity and composition between the two species, with *P. argentipes* harboring 18 phyla and *S. babu* containing 14 phyla. *Proteobacteria*, *Actinobacteria*, and *Firmicutes* dominated in both species, though each contained unique bacterial taxa that could potentially influence their biology. Notably, *P. argentipes* possessed unique phyla such as Fusobacteria, Armatimonadetes, Elusimicrobia, Chlamydiae, and Crenarchaeota, which are absent in *S. babu*. These interspecies variations underscore the potential role of gut microbiota in vector competence. Future research should focus on functional investigations of these identified bacterial communities to determine their specific roles in *Leishmania* development and transmission. Despite the limitations of our descriptive approach, this study contributes valuable insights into understanding the diversity of sandfly gut microbiota and establishes a foundation for future investigations into microbiota–vector–pathogen interactions.

## Figures and Tables

**Table 1:** Next-Generation Sequencing data of gut bacteria of *P. argentipes* (PAG-1) and *S. babu* (SBG-2) targeting the V3–V4 region of the 16S rRNA gene.

Parameters	*P. argentipes* (PAG-1)	*S. babu* (SBG-2)
No. of raw reads	378,038	321,964
No. of taxonomic assignments	629	641
Total Number of bases	113,789,438	96,911,164
Average read length (bp)	301	301
GC content (%)	55	55
Average read quality score	32.75	32.81

**Table 2:** Taxonomic classification of gut bacterial microbiota in *P. argentipes* and *S. babu*.

Taxa Level	*P. argentipes* (PAG-1)	*S. babu* (SBG-2)
Phyla	18	14
Classes	41	40
Orders	100	96
Families	181	108
Genera	315	327
Species	145	164

## Data Availability

All data presented in this manuscript are original. The 16S rRNA gene sequencing data have been submitted to the NCBI Sequence Read Archive. The dataset can be accessed through the following reviewer link: https://dataview.ncbi.nlm.nih.gov/object/PRJNA1266154?reviewer=bv5k4phi2gblv1jo2mlsoffufa (accessed on 3 July 2025). Further inquiries can be directed to the corresponding author.

## Supplementary Materials

The following supporting information can be downloaded at: https://www.mdpi.com/article/10.3390/microorganisms13071615/s1. Supplementary Figure S1: Stacked bar plot showing the relative abundance of bacterial species in *P. argentipes* and *S. babu* gut microbiota. The height of each colored segment represents the proportional abundance of individual bacterial species, allowing for direct comparison between the two sandfly species. Supplementary File S1: Krona plot output visualizing taxonomic hierarchy of gut microbiota in *P. argentipes*. Supplementary File S2: Krona plot output visualizing taxonomic hierarchy of gut microbiota in *S. babu.*
