# Supplementary material for: Metagenomic Analyses of Gut Bacteria of Two Sandfly Species from Western Ghats, India, Differing in Their Vector Competence for Leishmaniasis
Source: Microorganisms. 2025 Jul 9;13(7):1615. doi: 10.3390/microorganisms13071615 (PMC12300672; doi:10.3390/microorganisms13071615)
Supplement: Supplementary file 1 [file microorganisms-13-01615-s001.zip › Supplementary File S1_Krona_P_argentipes.html.html]

Javascript must be enabled to view this page.

magnitude
magnitudeUnassigned

PAG1\_krona

378038

0
1

1
0

0
1

1

368182
665

0
11

11

1

0
24

0
24

24
1

0
23

23

179
0

15

164
40

113

11

338
3

0
133

133
67

4
66

62

1

3

0
19

0
19

19

0
113

68
113

45

66
0

66
0

66

0
40

40

361
0

0
18

18
0

18

0
218

215

3
0

3

0
125

125

51450
36

1
130

0
56

56

73
30

34

0
9

9

0
23

23
12

2

1

8

12
51243

0
19

19
0

18
19

1

1173
51212

355
286

39
47

3

5

3

1

1

9

2

1

1
3

2

0
2

2

1

1
70

66
68

2

1

288
19

94
269

171

4

27653
32074

9

4412
4205

23

3

127

54

2

489
0

486
489

1

2

0
19

19

0
2

2

8017
0

8017
7689

49

51

228

1
1950

35

4

0
1

1

1909

21
9

11

1

2

15
0

15

1
2

1

16
18

2

8
9

1

6397
5586

1
19

18

237
40

197

2

2

37

1

0
2

2

158
78

7

37

36

134
310

45

131

42
43

1

40
38

2

0
22

22

5
0

5

99
106

6

1

30
4

3

21

2

18
1

14

2

1

62

2

8
2

6

15

0
4

0
4

4

14
0

0
14

14
0

14

2

0
52

0
52

52
0

52
0

52

0
7

7

29
0

29
0

0
29

0
29

29

30
0

30
0

0
29

29
4

5

20

1
0

0
1

1

0
20

0
3

3
0

3

10
0

10
0

0
6

6

1
4

3

3

3

1

0
1

1

10
0

0
10

10

9
0

0
9

9
1

3
8

5

47192
112

46860
12869

19694
33527

3
2

1

1848
77

12

1752
1192

306

175

4

64

11

1
0

1

6

1198
139

69

50

21

4

799
0

799

12
13

1

57

46

7125
9553

4

2110
1497

24

8

1

472

2

25

11

17

2

14

26

11

0
250

250

2
5

3

1

58
45

12

1

1

71
183

112

120
1047

908
551

44

1

305

2

2

1

2

1

4
0

4

1

13
4

1

7

1

88
396

20
3

17

29
0

29

0
19

1

3

15

8
27

19
10

1

1

7

104
27

23
8

15

2

52

9
109

69
64

1

4

31

0
6

6
0

6

0
62

6
62

56

98
1

2
0

2
0

2

0
1

0
1

1

34
94

0
1

1

0
1

1

14
6

1
0

1

7

5
2

0
2

2

1

0
28

2

26

1

0
1

1

0
9

5

4

0
122

122
0

122
118

2

2

0
15

8
0

8
7

1

7
0

7
0

7

5764
265501

1294
16

515
1179

3

2

654

0
1

1
0

1

2
0

2

2

40

0
12

3

9

0
2

2

1

4
0

4

3

25

3
0

3
0

2

1

0
1

1

1
7

2

3
4

1

1

66734
179871

28
27

1
0

1

50
3369

1242
1373

34
12

22

97

1660
1946

1

20

21

1
217

216

21

6

9

320
229

15

0
49

49

10

13
17

4

2564
113

112
117

5

2334
1860

15

37

270
301

7

3

7

14

8
5

3

86

27

2

27285
18280

129
292

7

34

115

1

6

1647
2734

0
151

151

905
775

130

16

1

14

1111
142

11
216

205

177

118

11

275

172
171

1

32
290

5

1

209

43
29

14

13
5

8

0
15

15

1451
454

9

967
988

21

54
55

1

530
66

464
181

5

264

6

8

88
68

9

11

201

204
356

152

1022
1869

9

91
331

240

260

0
1

1

241
246

5

6218
22

1475
5885

11

347
0

347

2
1

1

56
55

1

1

205

2

3

50

11

4

0
5

1

4

3713
3435

28

250

301
311

2

1

6

1

73342
66299

7043
4203

353

141
43

98

169
2038

1869

4

95
0

95

209

71805
7340

20
26

4
0

4

0
2

2

29056
1438

17615
296

8

17311
16954

185

5

20

6

17

3

4

85

32

902
10003

8204
8815

131

321

65

4

25

65

144

0
3

3

4

135

3
29

2

3
24

10

11

10
0

10

7
0

7
0

7
0

7

108
3

62
18

23

14
0

14

7

0
7

5

2

1

2
35

1

7

25

2
0

0
2

2

1
29140

28040
23637

10

7

64

3602
4194

19

152

421

20
7

13

15
30

15

46

5

15

12

1096
1099

3

0
2

1
0

1

0
1

1

27
15

2
7

3

2

5
0

5
1

3

1

1
20

1
19

7
0

7

11
1

10

1
13

12

24
8

16

0
20

5
7

2

13

152
66

31

55
48

1

6

4
244

1
2

1

3

1

0
2

2

6
4

2

14
23

4

5

20
21

1

0
7

7

2
175

7

1

2

2

161

0
5585

5585
4715

600
591

9

69

0
2

2

118
21

97

4

43
0

43

1

6
4

2

8

6

4

1
2

1

7

1
0

0
1

0
1

1

1
0

1

6765
1805

10
0

10
0

10

3

94

14
8

5
6

1

0
1

1

1

3
0

3

0
35

30
35

2

1

1

1

36
0

36
27

8
6

2

1

185
4730

747
884

37

6
5

1

94
71

23

1862
132

2

10

1

1709

1

7
0

7

1597
1395

28

14

1

4

1
2

1

1

2

2

2

8
2

6

1

1

1

113
116

3

6

12

1

202
129

2

71
70

1

0
3

3
1

2

27

3

0
23

0
23

2
23

0
21

21

2144
7

37

26
0

26
15

0
2

2

1

1

0
7

5
7

2

397
0

3
397

1
394

393
106

287

0
27

27
1

25
26

1

0
1368

1368
14

0
1091

1091
1081

10

263
10

1

252

0
282

282
2

278
79

2

10

1

186

2

21
0

21

57
1

1

3
0

3

15

0
37

16
0

16
0

16

20
0

0
20

20

0
1

1

9855
