# Supplementary material for: Metagenomic Analyses of Gut Bacteria of Two Sandfly Species from Western Ghats, India, Differing in Their Vector Competence for Leishmaniasis
Source: Microorganisms. 2025 Jul 9;13(7):1615. doi: 10.3390/microorganisms13071615 (PMC12300672; doi:10.3390/microorganisms13071615)
Supplement: Supplementary file 1 [file microorganisms-13-01615-s001.zip › Supplementary File S2_Krona_S_babu.html.html]

Javascript must be enabled to view this page.

magnitude
magnitudeUnassigned

SBG2\_krona

321963

689
311993

0
39

39

243
0

1
0

0
1

1

209
0

208

0
1

1

0
33

33

12
2

6

4

0
22

22

63697
22

17
0

0
17

17
0

9
17

8

0
8

8

0
1

0
1

1

63536
14

0
2

0
2

2

63520
1511

0
52

1
2

1

50

17

20
0

20

13
0

13

0
30

30

1
2

0
1

1

1
0

1

1

43
148

1

4

71

29

9130
0

9130
8789

183

73

85

2

7956
8943

34
35

1

22
0

22

59

1

84
207

72

41

10

2

24
190

166

471
188

88

195

22
0

2

20

1
3

1

1

2
30

28

2
1

1

15
2

13

6
1

5
4

1

151
116

31

0
2

2

2

358
0

355
358

1

1

1

322
25

107
293

3

183

4

51
16

22

13

279
201

0
2

2

5
1

4

1
0

1

1
0

1

2

13

35
46

3

4

4

1
0

1

1

2

4

5
2

3

19

32
28

2

1

1

6
152

19

2

9

116

2
3

1

42200
36914

7

5279
5022

154

8

71

20

4

0
59

23
59

2

34

49
0

0
49

49
0

49

0
5

4
5

1

84
0

0
11

0
1

1

10
0

10
0

10

27
1

0
16

16

0
10

10

11

0
17

2

15
7

7
8

1

18

1

6121
182279

837
7

1

381
673

8

280

3

1

1

0
2

2

1

1
0

1

104

0
1

1

14
0

14

28

4

7205
1699

42

0
29

29
23

5
6

1

2
1

1
0

0
1

1

0
5

5
4

0
1

1

0
25

18
25

5

2

5373
243

2213
1939

0
1

1

14

2

1

1

1

1

8

3
5

2

38

5
6

1

1

1

1

161
155

6

2

1

4

3

4

5

1
5

4

1

3
6

3

1

883
1002

61
85

24

30

3

1

109
1668

2

6

8
0

8

2

1541

156
247

89

2

14

4
0

4
2

2

0
12

0
12

12

1
0

1
0

1

11519
76743

2
0

0
2

2

5
35

16
30

2
12

10

2
0

2

22988
2

637
611

25
26

1

22349
18620

19
7

12

29
12

17

10

2

7

44

3020
3522

24

182

296

1

17

70

8

27
13

5
1

4
2

2

9
4

2

3
2

1

1
0

1
0

1

0
6797

6797
5722

1

79
77

2

8

3
0

3

1

2
3

1

95
14

81

13

11
7

4

7

0
59

59

791
783

8

3

1

96
12

0
1

0
1

1

11
42

10

16
17

1

0
4

4

2

3
0

2

1

8

28
0

8

18

2

0
24

24

5
16

11

388
85

3
207

2

2

2

2

192

2

2

5
4

1

0
1

1

40

4
1

3
0

3

8
7

1

3
0

3

12
4

8

23
0

21
23

2

3

34676
1164

7123
694

5

4
0

4

220

176

5447
6023

100

88

6

13

52

317

1

26389
271

25603
26111

14

215

14

7

29

26

159

38

6

4

3

0
1

1
0

1

6
34

7
9

2

19
6

8

5

8
102

11
14

3

70
80

9

1

34
19

13
0

13

2

91372
33013

1839
124

8

7

1675
7

1668
1663

5

6
25

19

7

3

8

8
3408

3290
1101

67

4

177
0

177

1

11

1

3

97

10
6

4

54

696
1763

14

1053

1

106
110

2
1

1

2

54
1722

57
55

2

1259
1611

3

67

36

5

6
3

3

11

209
224

2

2

11

1

58
1659

585
683

2

43

3
52

49

1

918
443

1

19

9

443
0

443

2

1

14370
9211

950
107

139

171
184

13

170

118
119

1

228
10

218

3

96
167

71

293
687

129

0
1

1

132
37

95

17
16

1

115

1
4

3

7
1

6

119
51

1

9

2

4

50

2

7
212

13
8

5

151

31

10

142

31
30

0
1

1

515
87

428
240

6

1

31

150

1181
1728

0
65

65

443
357

86

29

10

21
36

6

7

2

561
174

5

371
382

11

31899
35342

3443
2070

0
38

38

76

163

92
1040

948

50
13

37

6
5

1

40
0

0
6

6

8

0
26

0
23

0
23

23

3
0

0
3

3

0
5

5
0

5
0

1
5

4

162
0

162
44

2

103

13

18
0

2
0

2

0
16

16
0

0
16

16

31
0

21
0

0
21

13

8

10

0
32

32
0

0
3

3
0

3

0
29

0
29

29

0
2908

556
0

556
2

0
554

554
234

3

317

23
0

23
4

17
10

6

1

0
2

2

214
0

214
0

214
71

134

8

1

0
2060

2060
12

1557
0

1557
1553

4

2

11

55
478

5

67

351

26

0
9

9
0

9

0
20

20
0

20
11

9

153
61580

114
0

40
112

3

3
0

2

1
0

1

9
13

1

2

1
0

1

11
3

6

2

1
16

15

26
0

26

1
0

1
0

1

1

20580
61194

0
4

4
0

4

132
718

2
34

32
31

1

37
0

36

1

160
27

3

19
38

19

7

85

35
2

33

4
231

147
152

4

1

75

12
89

2
1

1

56
75

1

3

8

1

6

79
0

79
5

74

39813
25824

3
2

1

1205
173

14
16

2

20

61

46

89

723
0

723

2
0

2

75

51
1038

5
2

3

1

59
63

3

1

918
496

354

64

2

1

1

2164
121

6

2032
1353

18

4

1

198

60

398

5

9401
6647

6
5

1

0
342

342

9

37
55

18

2338
1844

11

2

1

1

362

2

23

21

17

32

3

1

5

13

2
0

2

1

1

1

1
0

1

0
4

4

1
0

1

171
124

47

119
0

119
0

117
119

2

47
0

47
0

0
47

0
47

47

1

0
101

0
26

26
0

26

14
0

14
0

14
6

8

0
3

3

58
0

58
0

58

2

9970
