# Supplementary figures and images for: Metagenomic Analyses of Gut Bacteria of Two Sandfly Species from Western Ghats, India, Differing in Their Vector Competence for Leishmaniasis
Source: Microorganisms. 2025 Jul 9;13(7):1615. doi: 10.3390/microorganisms13071615 (PMC12300672; doi:10.3390/microorganisms13071615)

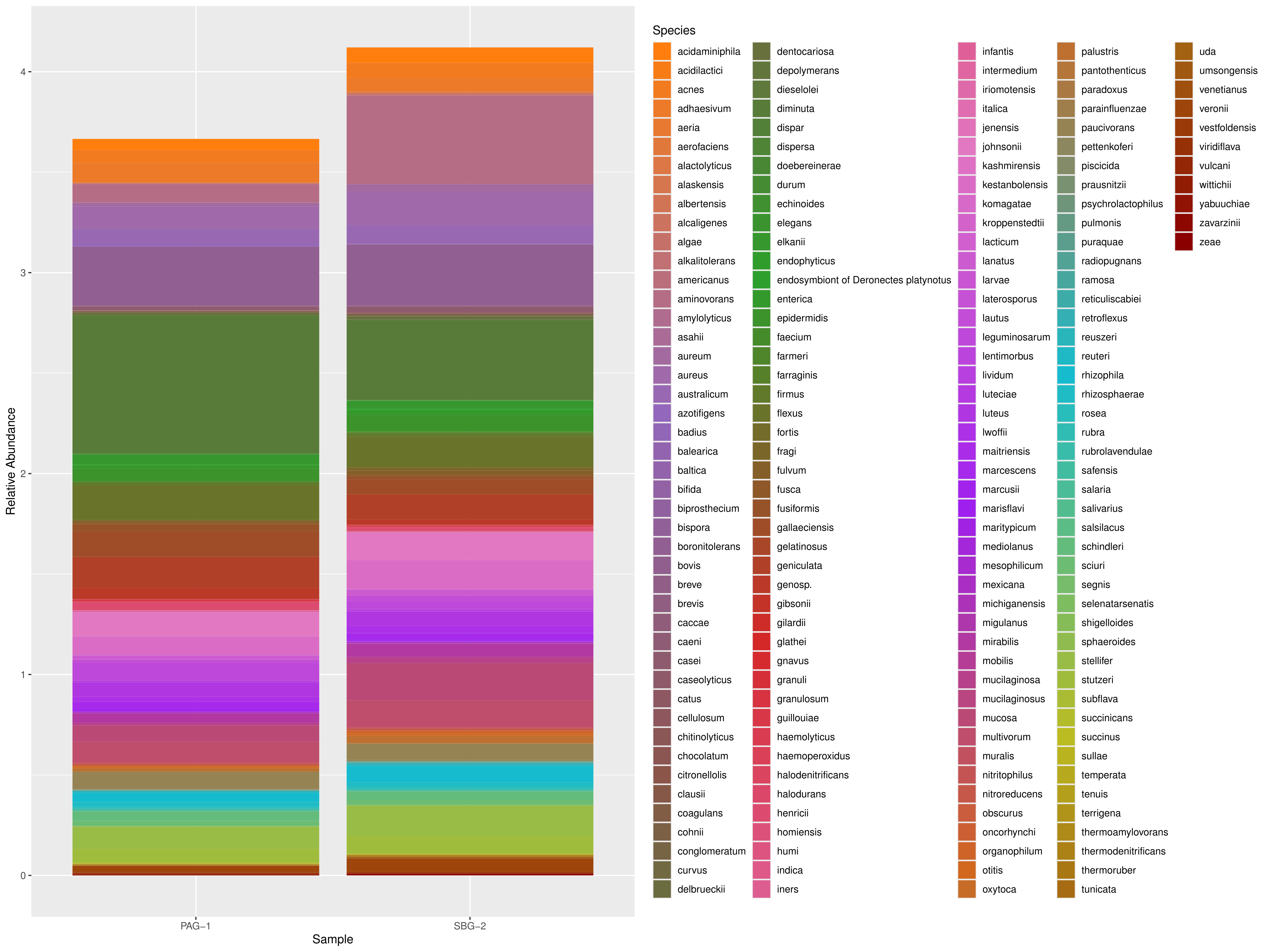

Supplement: Supplementary file 1 [file microorganisms-13-01615-s001.zip › Supplimentary Figure S1.tif]
